# Lack of Healthy Food Options on Children’s Menus of Restaurants in the Health-Disparate Dan River Region of Virginia and North Carolina, 2013

**DOI:** 10.5888/pcd12.140400

**Published:** 2015-03-26

**Authors:** Jennie L. Hill, Nicole C. Olive, Clarice N. Waters, Paul A. Estabrooks, Wen You, Jamie M. Zoellner

**Affiliations:** Author Affiliations: Nicole C. Olive, Paul A. Estabrooks, Wen You, Jamie M. Zoellner, Virginia Tech, Blacksburg, Virginia; Clarice N. Waters, National University of Singapore, Singapore.

## Abstract

**Introduction:**

Interest has increased in understanding the types and healthfulness of restaurant foods for children, particularly in disadvantaged areas. The purpose of this community-based participatory research study was to describe the quality of restaurant food offered to children in a health-disparate region in Virginia and North Carolina and to determine if the availability of healthy foods differed by location (rural, urban) or by the predominant race (black, white, mixed race) of an area’s population.

**Methods:**

Restaurants offering a children’s menu in the 3 counties in Virginia and North Carolina that make up the Dan River Region were identified by using state health department records. Research assistants reviewed menus using the Children’s Menu Assessment (CMA), a tool consisting of 29 scored items (possible score range, −4 to 21). Scores were calculated for each restaurant. We obtained information on the predominant race of the population at the block group level for all counties from 2010 US Census data.

**Results:**

For the 137 restaurants studied, mean CMA scores were low (mean, 1.6; standard deviation [SD], 2.7), ranging from −4 to 9 of 21 possible points. Scores were lowest for restaurants in the predominantly black block groups (mean, 0.2; SD, 0.4) and significantly different from the scores for restaurants in the predominantly white (mean, 1.4; SD, 1.6) and mixed-race block groups (mean, 2.6; SD, 2.4) (*F* = 4.3; *P* < .05).

**Conclusion:**

Children’s menus available in the Dan River Region lack healthy food options, particularly in predominantly black block groups. These study findings can contribute to regional efforts in policy development or environmental interventions for children’s food quality by the community-based participatory research partnership and help local stakeholders to determine possible strategies and solutions for improving local food options for children.

## Introduction

Reversing trends in childhood obesity prevalence is a public health priority because of the negative physical and mental health effects the condition confers across the lifespan (1–3). The high prevalence of childhood obesity has been concurrent with a change in the extent to which children consume foods away from home (4,5). Recent literature suggests that over one-third of children and adults eat food away from home on any given day (4). Furthermore, those that eat food away from home consume approximately 200 more kilocalories per day, regardless of whether the restaurant is fast food or full service (4,5). These consumption patterns concur with disparities in childhood obesity across racial/ethnic groups in that African Americans consume more of their calories away from home than other racial groups (5,6).

To complement health education and social marketing efforts, policy and environmental strategies are needed to reduce childhood obesity through dietary changes (7). Residents of minority, low-income neighborhoods and rural areas are more likely to have poor access to grocery stores and greater access to and consumption of restaurant food (8–12). To date, only a few studies (4,6) have examined the degree to which fast food and full-service restaurants provide healthy menu options, and even fewer studies focus specifically on children’s menus (5,13). Those that do found that children’s menus often did not include a variety of healthy options (5,13). The purpose of this study was to examine the food environment by assessing children’s menu options at restaurants in the Dan River Region of south-central Virginia and north-central North Carolina, on the basis of rural and urban areas and by race at the census block group level.

## Methods

### Study area

The Dan River Region, a predominantly rural area of south-central Virginia and north-central North Carolina, comprises 3 counties covering nearly 1,800 square miles with a population of 137,000 (14). The region is anchored by a small regional city (population, 43,000; area, 44 sq mi). The region is designated a “medically underserved area/medically underserved population” by the Health Resources and Services Administration (15) and is characterized by low educational attainment and high unemployment among adults (14, 16-17). In the regional city approximately 41% of children aged 17 years or younger live in poverty compared with 15% of children in Virginia overall (17). Prevalence of chronic diseases such as diabetes and obesity is high among adults (14, 16–17). Although regional data on childhood obesity are limited, data collected by school nurses in 1 local school district showed 17% of first-graders were overweight (ie, body mass index [BMI] [kg/m^2^], 85th–94th percentile) and 19% were obese. By fifth grade, in this same cohort, prevalence increased to 19% overweight and 36% obese.

### Community-based approach

Given the high prevalence of obesity and obesity-related chronic conditions in adults and children in the Dan River Region, community stakeholders and research partners came together in 2009 to form a community–academic partnership, the Dan River Partnership for a Healthy Community (DRPHC) (18). This partnership operates under the principles of community-based participatory research (19). Through a participatory process, the DRPHC prioritized potential areas for interventions and programs related to obesity and related health conditions (18). To date, the DRPHC has pursued initiatives to address obesity among adults through physical activity programs and among children through community gardens and a pediatric weight-loss program.

Community stakeholders also recognize that the built environment is an important factor to consider when designing obesity-reduction programs, but no regional data existed on the built environment or the food environment. Thus, the DRPHC initiated a series of built-environment studies to determine access to and availability of healthy food and physical activity opportunities (20,21). Because racial equity and differences between rural and urban residents in the region are a major DRPHC focus, priority was given to examining children’s menus on the basis of these 2 focus areas. This study was conducted as part of a larger, ongoing initiative to quantify the food environment and determine local access and availability of healthy food in restaurants in the Dan River Region and focused specifically on the offerings on children’s menus.

### Data collection

#### Classifying restaurants

As part of the larger ongoing DRPHC food environment initiative, all restaurants within the region were classified by type and systematically audited using the Nutrition Environment Measures Survey–Restaurants (NEMS–R) (Figure) (22). Restaurants included fast-casual, fast-food, sit-down, and specialty restaurants (23). To focus on foods for children, all restaurants with children’s menus (n = 137) were further audited with the Children’s Menu Assessment (CMA) tool and are included in this study (24). Details of enumeration methods and data collection process are provided elsewhere (25). In 2013 research staff members visited each restaurant with a children’s menu and reviewed the menu on site. There were no statistical differences by type of restaurant (eg, fast-food, sit-down) that offered a children’s menu.

**Figure 1:**
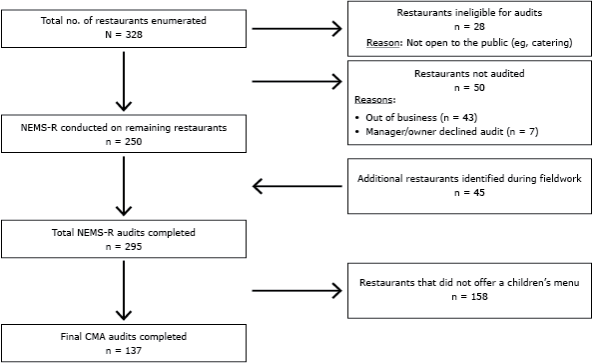
Restaurants with a children’s menu audited using the children’s menu assessment (CMA) tool. The Nutrition Environment Measures Survey–Restaurants (NEMS-R) was used to conduct audits of restaurants (19,20).

### Assessment of children’s menu

The CMA is an expansion of NEMS-R and captures additional information on children’s menus (24). The initial CMA calibration study by Krukowsi et al. demonstrated high interrater reliability and test–retest reliability (24). For this study, research assistants with training and certification for the NEMS-R tool (23) received specialized training on the CMA tool. Pairs of auditors rated all children’s menus at each restaurant, and the mean κ coefficient of 0.946 (range 0.63–1.0) indicated high interrater reliability.

The CMA consists of 29 items total (24). Of the 29 items, 21 are scored menu items (ie, availability of healthy or less healthy items) and 8 are descriptive items (ie, restaurant type, cuisine type). To be scored, menus must provide sufficient detail and description of items to classify them as healthy. Similar to the NEMS-R tool, the CMA classifies items as healthy based on US standards such as the 2010 Dietary Guidelines for Americans and the US Department of Agriculture’s standards (23,24). Possible scores range from −4 to 21, with higher scores indicating greater availability of healthy menu options for children. Per the published CMA protocol (24), points (0, 1, or 2) are given for menus based on the number of healthy entrees, salads, and whole grains offered and whether healthy beverages, side dishes, desserts, and salad dressing are offered. One point is deducted (−1) for soda, free refills on sugary beverages, unhealthy desserts, and the use of toys or other child-directed marketing (24). This study reports 4 children’s menu scores, including overall CMA score, overall CMA score excluding toy/marketing deduction, healthy entrée only, and whole grain option only.

### Geographic location

To compare differences in children’s menu options based on location of the restaurant, we categorized restaurants as urban (within the regional city) or rural (outside the city limits) (20,21).

#### Block group-level data

For all counties, data on race/ethnicity at the block group level were obtained from the 2010 US Census (14). The 2010 census data divides the region into 157 block groups; nearly 95% of the region’s population is black or white (14). On the basis of previous sensitivity analyses, block groups with more than 55% of a single race were classified as predominantly white or predominantly black, whereas block groups with less than 55% of a single race were classified as mixed-race block groups (20,21). Restaurants with a CMA score were geocoded and assigned a block group race accordingly.

### Statistical analysis

Given the high interrater reliability among auditors, a random-delete strategy was used to eliminate 1 audit for each restaurant to obtain the final data set for scoring and analysis. Descriptive statistics were calculated for CMA items such as nutrition labeling (ie, nutrition information for menu items provided), entrees offered, beverages, and toys–marketing (Table 1). One-way analysis of variance (ANOVA) tested for mean differences in healthy-food availability scores by restaurant type (eg, fast food), urban or rural location, and block group race; we used Student–Newman–Keuls test post hoc when 2 or more groups were compared. Data was analyzed using SPSS version 20.0 (IBM Corp), and statistical significance was set at *P* < .05.

**Table 1:** Description of Children’s Menu Assessment Categories Scored on Children’s Menus (N = 137), Restaurants in the Dan River Region of Virginia and North Carolina, 2013

Children’s Menu Assessment Categories	N (%)
**Nutrition guidance**	
Any nutrition information	2 (1.5)
Symbol indicating healthy item	15 (10.9)
**Entrees**	
Healthy entrée	15 (10.9)
Healthy entrée salad	3 (2.2)
Whole-grain option	9 (6.5)
**Beverages**	
Juice, any	55 (40.1)
Juice listed as 100% juice	20 (14.6)
Milk, any kind	68 (49.6)
Milk listed as low-fat, 1%, or nonfat	36 (28.5)
Soda (default option)	39 (28.5)
Free soda refill	10 (7.3)
Substitution allowed for healthier beverage	45 (32.8)
**Side dishes**	
Nonfried vegetables	12 (8.8)
Fruit, any	54 (39.4)
Fruit without added sugar	32 (23.4)
Dairy side dish	1 (0.7)
Substitution allowed for healthier side dish^a^	30 (21.9)
**Desserts**	
Healthy desserts^b^	0
Dessert included with children’s meal	10 (7.3)
**Toys/marketing**	
Branded marketing to children^c^	11 (8.0)
Toy included with child’s meal	30 (29.1)

a Customer can substitute a healthy side dish, such as carrot sticks or apples, for french fries.

b Healthy desserts are defined by the CMA protocol as those low in fat, sugar, or calories (eg, low-fat frozen yogurt).

c Branded marketing to children (eg, pop culture references or movie characters).

## Results

We found 328 restaurants in the region. Of these, 78 were ineligible (28 were not open to the public, and 50 had either gone out of business or their managers declined to be audited), leaving 250 restaurants eligible for audit. During field work, an additional 45 eligible restaurants were found for a total of 295 restaurants, which we audited using NEMS–R. Of these, 137 restaurants had children’s menus and were audited with the CMA tool. Seventy-six (56%) were urban (12 fast casual; 29 fast-food; 35 sit-down). The remaining 61 (44.0%) were rural (6 fast-causal; 22 fas- food; 33 sit-down). Overall, 18 (13.0%) were fast-casual restaurants, 51 (37%) were fast-food restaurants, and 68 (50%) were sit-down restaurants. Most restaurants (84 [61%]) did not specify an age range for the children’s menu; 29% specified that their children’s menu was for children 12 years old or younger, and 9%, for children 10 or younger.

On average, the restaurants offered 5 (SD, 2.0) entrees on the children’s menu, but only 15 of the 137 (11%) included at least 1 healthy entrée (range, 0–4) among entrees offered. Twelve (9%) offered a nonfried vegetable side item, and 54 (39%) offered fruit, although only 32 (23.4%) offered fruit without added sugar. Only 10 (7.3%) of the 137 included a dessert as part of the children’s meal; however, no restaurants offered healthy desserts (eg, low-fat ice cream). Thirty restaurants (29%) included a toy in the children’s meal, and 11 (8%) used branded marketing to promote their children’s meal. In addition, 39 (29%)of the 137 restaurants specifically listed soda as the beverage option on the children’s menu, and only 45 (32.8%) offered a healthier beverage substitution in lieu of a sugar-sweetened beverage. Although 68 (50%) of the restaurants offered milk, only 36 (28.5%) offered low-fat or skim milk as a beverage option for children. Additionally, 55 restaurants (40%) offered juice drinks as a beverage option for children, but only 20 (15%) offered 100% juice as an option (Table 1).

Overall CMA scores ranged from −4 to 9 with a mean score of 1.6 (SD, 2.7) (Table 2). The mean CMA score excluding deductions for the branded marketing and toys was 1.85 (SD, 2.9) ranging from −3 to 11. The score for the subscale healthy entrée was 0.02 (SD, 0.58) and for whole grain was 0.13 (SD, 0.50). The overall CMA mean score differed by restaurant type (*F* = 22.4, *P* < .001), and post hoc analyses demonstrated that fast-casual restaurant scores (5.0; SD, 3.7) were significantly higher (*P* < .05) than fast-food (1.3; SD, 2.2) and sit-down restaurant scores (0.8; SD, 2.1).

**Table 2:** Mean Scores, Children’s Menu Assessment and Test for Differences by Location, Restaurants in the Dan River Region of Virginia and North Carolina, 2013

Children’s Menu Assessment Score	All, N = 137, Mean (SD)	Urban, n = 76, Mean (SD)	Rural, n = 61, Mean (SD)	*P* Value
Overall score^a^	1.55(2.73)	1.78(2.93)	1.28(2.46	0.291
Overall score (excluding toy)	1.85(2.93)	2.16(3.11)	1.48(2.68)	0.177
Healthy entrée score	0.02(0.58)	0.29(0.69)	0.08(0.38)	0.037
Whole grain	0.13(0.50)	0.05(0.32)	0.23(0.64)	0.038

a Children’s menu assessment scores can range from −4.0 to 21.0.

Overall CMA scores with and CMA scores without the point deductions for toys and other child-directed marketing were not significantly different between the 76 urban restaurants and the 61 in rural areas (Table 2). The healthy entrée ratio score was higher in urban areas than rural areas (*F* = 4.45, *P* < .05). On the other hand, the whole-grain ratio score was higher in rural than in urban areas (*F* = 4.39, *P* < .05). Nutrition labeling in restaurants in urban and rural areas was also compared but was not significantly different by location. 

Predominantly white block groups had the highest number of restaurants per block group (34), predominantly black block groups had 12, and mixed race block groups had 6. Overall CMA scores were lower in the predominantly black block groups (0.15, SD 0.44) than in the predominantly white block groups (1.38, SD 1.61) or the mixed race block groups (2.63, SD 2.44), with significant differences among predominantly black and mixed block groups (*F* = 4.3, *P* < .05). There were no significant differences for the healthy entrée ratio score (*F* = 0.40, *P* = .68) or whole-grain ratio (*F* = 0.19, *P* = .83) by block group race.

## Discussion

Our study examined availability of healthy options on children’s menus in a predominantly rural, health-disparate region. Regardless of restaurant type, the healthy-food availability scores for the children’s menu were low (mean, 1.9; SD, 2.9; range −3 to 11) on a 21 point scale. Similar to findings of Kurkowski et al, the overall CMA scores in this study were within the lower third of the range (24,25). Furthermore, one-third of the restaurants for both studies listed soda as a drink option on their children’s menu, and even fewer offered 100% juice. This finding is of concern given the association between sugar-sweetened beverage consumption and children’s obesity risk (26, 27), particularly for those children who consume more food away from home than at home (28).

Overall CMA scores of restaurants in urban and rural locations were not different; however, there were differences regarding the healthy entrée and whole-grain options. Urban areas had higher scores for healthy entrée options (eg, grilled chicken instead of breaded), whereas rural areas had higher scores for whole grain.

Our results demonstrate that the overall number of restaurants and the average CMA scores were significantly lower in predominantly black block groups than in white and mixed block groups. This result aligns with the findings of other studies that demonstrate lower availability of healthy food options in minority neighborhoods or areas (8–12,24). As previously noted, blacks eating food away from home consumed more calories than other racial groups also consuming food away from home (6). It was clear from our study that CMA scores were low throughout the region and significantly lower in black block groups than in white and mixed-race block groups. For families who make food choices away from home, this is a concern.

This study has limitations. It cannot be determined whether parents ordered from the children’s menu for their children because a restaurant offered such a menu. Also, lack of nutritional information and product descriptions on several menus made it difficult to determine healthfulness of menu items. This lack of information may lead to misclassification of some items; however, it does reflect the consumer’s experience in trying to determine a healthy item based on the menu description (24). Children’s menus were offered at fewer than half (46%) of the restaurants found in this region. We found, in another study, low availability of healthy options at all restaurants, not just on the children’s menus (21). Finally, the number of restaurants in the region with a children’s menu, 137, may limit our ability to detect statistical differences. This is the number of the restaurants in this region that offered a children’s menu, not a selected representative sample; therefore, the data do present what is available and marketed to children in the region through children’s menus.

This study provides additional information to support the use of CMA in evaluating an aspect of the food environment, children’s menus (24,25,29). It would also be advantageous to investigate the purchasing behavior of adults and children to see whether the food on children’s menus is what is typically purchased. This study’s findings show that healthy food options are limited on most of the region’s children’s menus. As with data from another community-based study (30), these data could be used to encourage community stakeholders and local food establishments to consider improvements to the quality of food on children’s menus.

The Dan River Region is a rural, health-disparate region with a high prevalence of obesity among both adults and children. The findings from this child-focused study support previous studies in the region that demonstrate an overall lack of healthy food options (21). Effective, comprehensive approaches to the individual and environmental factors contributing to obesity are urgently needed. 
